# Conservative Treatment of an Isolated Greater Tuberosity Fracture With Dislocation: Management of Post-Traumatic Subacromial Impingement Syndrome and a Mini-Review of the Literature

**DOI:** 10.7759/cureus.67267

**Published:** 2024-08-20

**Authors:** Muhammed Yusuf Afacan, Cumhur Deniz Davulcu

**Affiliations:** 1 Department of Orthopaedics and Traumatology, Istanbul University-Cerrahpasa, Cerrahpasa Faculty of Medicine, Istanbul, TUR

**Keywords:** subacromial impingement syndrome, subacromial bursitis, diagnostic shoulder ultrasound, transcutaneous electric nerve stimulation (tens), shoulder sling, conservative treatment, non-displaced fracture, proximal humerus fracture dislocation, greater tuberosity fracture dislocation

## Abstract

This case report evaluates the effectiveness of conservative treatment for an isolated greater tuberosity fracture-dislocation, detailing the treatment process and addressing post-traumatic subacromial impingement syndrome with a mini-review of the literature.

A 26-year-old male fell from a height, resulting in a self-reduced dislocated shoulder. Examination revealed extensive ecchymosis, pain, and limited motion in the right shoulder. Radiological assessments showed an isolated greater tuberosity fracture, partial tears of the supraspinatus and subscapularis muscles, a suspected ALPSA lesion, and periarticular effusion. Initial treatment included a shoulder sling, passive elbow and wrist exercises, and pendulum exercises starting in the second week. At six weeks, persistent pain prompted TENS therapy and advanced rehabilitation exercises. At three months, the patient continued to experience pain and restricted shoulder movement. An MRI ruled out labral pathology, and a shoulder ultrasound revealed post-traumatic subacromial bursitis, leading to a diagnosis of subacromial impingement. A diagnostic ultrasound-guided injection of prilocaine into the subacromial bursa significantly improved the range of motion and alleviated pain within one hour. Treatment recommendations included avoiding overhead activities, NSAIDs, and continued rehabilitation. By six months, the patient had achieved a pain-free range of motion of 180 degrees.

This case demonstrates that conservative treatment and appropriate rehabilitation can effectively manage isolated greater tuberosity fractures and associated glenohumeral joint dislocations. Early diagnosis and suitable rehabilitation strategies for post-traumatic subacromial impingement syndrome positively influenced the patient's recovery. Given the patient's youth and swimming background, steroid injections were avoided due to potential complications, with successful recovery achieved through NSAIDs, overhead activity restriction, and rehabilitation.

## Introduction

Proximal humerus fractures are common [1], but isolated greater tuberosity (GT) fractures are relatively rare [2,3], and greater tuberosity fractures accompanied by glenohumeral joint dislocation are even more infrequent. The treatment methods and outcomes of isolated greater tuberosity fractures and their associated dislocations are subjects of ongoing debate in the literature. The degree of displacement of the GT fractures is crucial in the decision-making process for treatment [1]. A superior displacement of the GT by 2 mm increases the force required for abduction and can lead to subacromial impingement [1,2,4,5]. Superior displacement greater than 5 mm is associated with the severity of rotator cuff damage [6], and in such cases, surgical intervention with anatomic reduction and internal fixation is recommended [2]. If anatomical reduction is not achieved, the displaced greater tuberosity narrows the subacromial space, leading to subacromial impingement syndrome [2]. Post-traumatic subacromial impingement syndrome can result in frequent shoulder pain and restricted joint mobility during activity. Conservative treatment is recommended for fractures with less than 5 mm of displacement [3,7].

This case report aims to evaluate the effectiveness of conservative treatment in a case of isolated greater tuberosity fracture-dislocation, discuss the treatment process, manage post-traumatic subacromial impingement syndrome, assess treatment outcomes, and provide a literature mini-review based on this case.

## Case presentation

**Figure 1:**
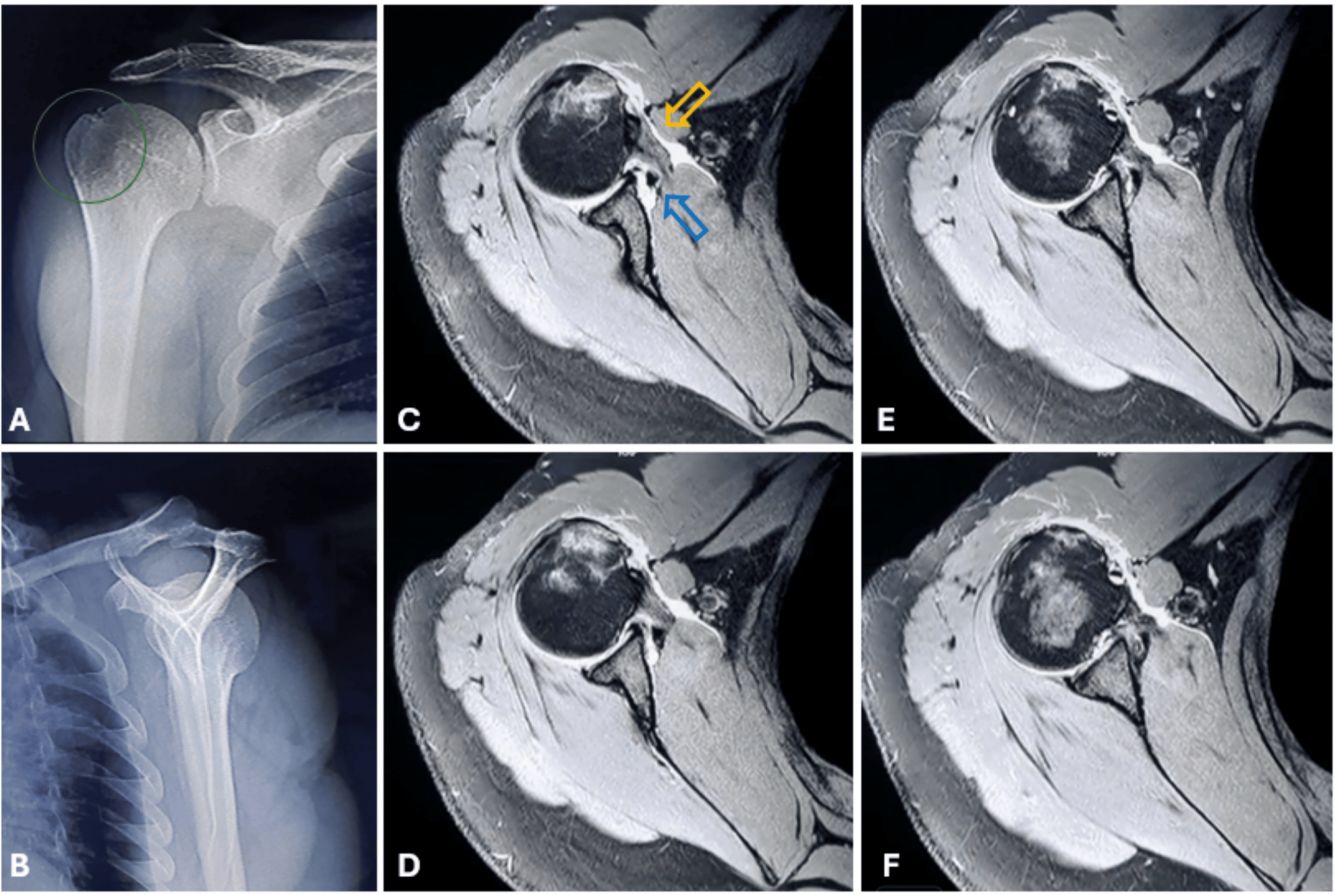
Imaging studies of a patient with a right greater tuberosity fracture after falling from a height and hanging in mid-air by his right shoulder. A and B are direct radiographs, and C, D, E, and F are consecutive axial T2-weighted MRI images. Figure 1A: True anteroposterior radiograph showing the right shoulder with a non-displaced greater tuberosity fracture (green circle). Figure 1B: Scapular Y radiograph highlighting normal alignment. Figures 1C-1F: Consecutive T2-weighted MRI sequences in axial sections displaying partial subscapularis tear (orange arrow), suspected ALPSA lesion (blue arrow), bone marrow edema, and widespread periarticular effusion.

**Figure 2:**
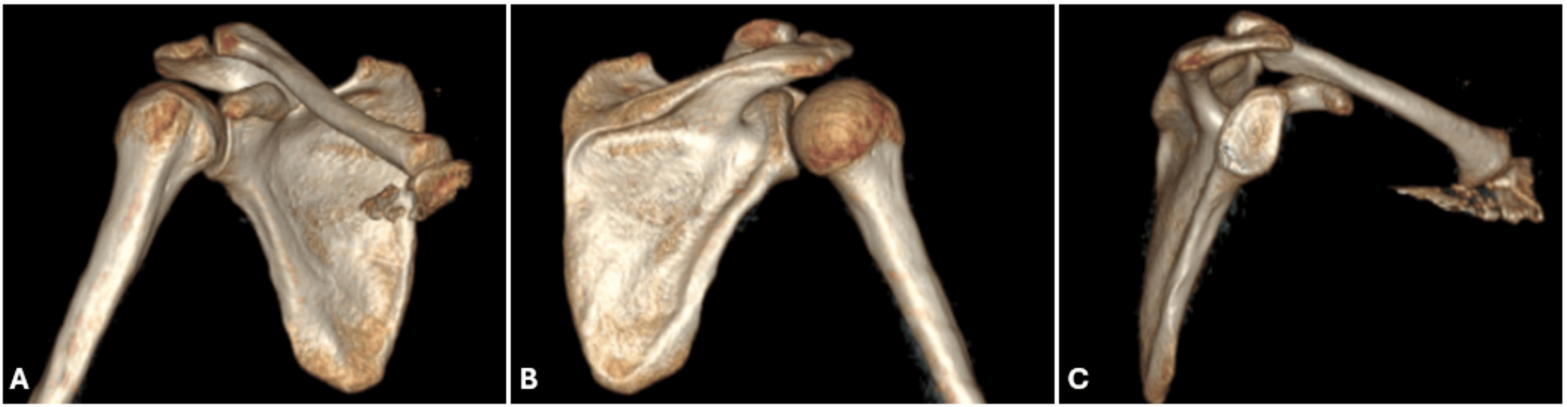
Three-dimensional computed tomography reconstructions of the right shoulder demonstrate an intact glenohumeral joint in the anterior (A) and posterior (B) views and an intact glenoid after the humerus was distracted (C).

A 26-year-old male patient presented to the clinic after falling from a height and hanging in mid-air by his right shoulder. During the incident, the patient’s shoulder dislocated and spontaneously reduced while he was being extracted from the trauma site. Physical examination revealed widespread ecchymosis, pain, and restricted movement in the right shoulder region. Forward flexion and lateral elevation were painful and limited to 20 degrees, and external rotation was completely restricted and painful. Hypoesthesia was noted in the shoulder region, but no neurovascular deficits were detected. Direct radiographs revealed an isolated greater tuberosity fracture (Figures 1A, 1B). Since this was the patient's first dislocation, a computed tomography (CT) scan was performed to evaluate the bony structure, particularly the glenoid (Figure 2). The glenoid was intact. Due to the trauma mechanism, magnetic resonance imaging (MRI) of the right shoulder was conducted to assess the rotator cuff. Imaging revealed an isolated greater tuberosity fracture, a partial supraspinatus tear, a partial subscapularis tear, a suspected anterior labroligamentous periosteal sleeve avulsion (ALPSA) lesion, and extensive fluid accumulation around the joint (Figures 1C-1F).

**Figure 3:**
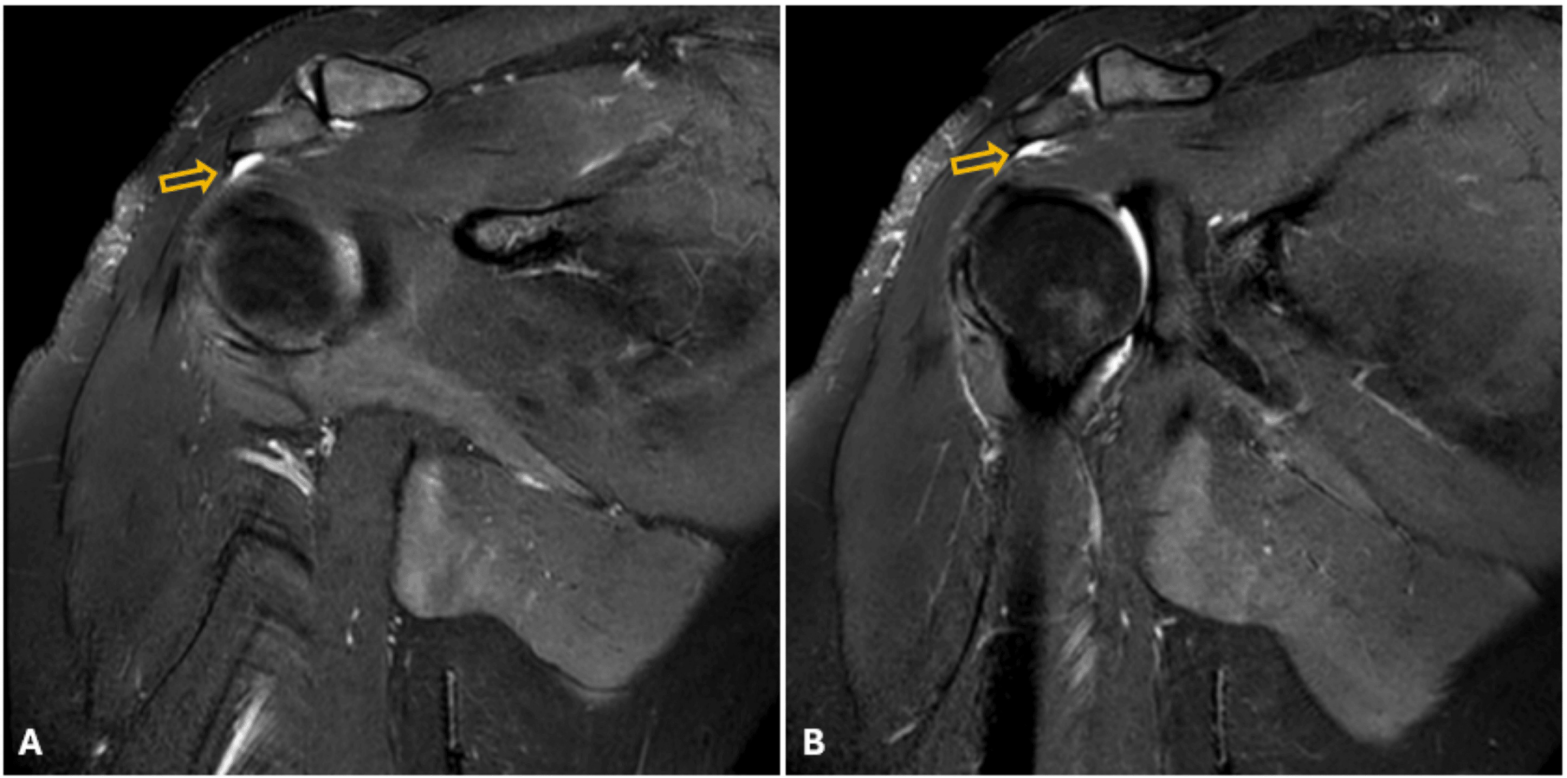
Coronal section magnetic resonance imaging (MRI) images of the right shoulder, indicating suspicion of subacromial bursitis in consecutive sequences A and B. The orange arrow shows the region of bursitis with an increased signal.

Conservative treatment was recommended because the displacement of the greater tuberosity was less than 5 mm. The patient was fitted with a shoulder-arm sling and initiated passive elbow and wrist exercises. In the second week, shoulder pendulum exercises were started. In the sixth week, due to persistent pain, the patient began receiving conventional transcutaneous electrical nerve stimulation (TENS) therapy three times a week (20 minutes, 1.5W/cm²). Additionally, scapular stabilization exercises, isometric shoulder girdle strengthening exercises (with the arm at the side), followed by isotonic strengthening exercises, core stabilization exercises, and proprioceptive and plyometric exercises, were initiated. At the three-month follow-up, the patient continued to experience pain and restricted movement in the right shoulder. The Hawkins test and Neer sign were positive, and the Jobe test, bear hug test, belly-press test, and lift-off tests were negative. A follow-up MRI was performed due to the suspicion of ALPSA. The glenoid and labrum appeared intact. An ultrasound of the right shoulder revealed post-traumatic subacromial bursitis consistent with the MRI findings (Figure 3), leading to a diagnosis of subacromial impingement.

**Figure 4:**
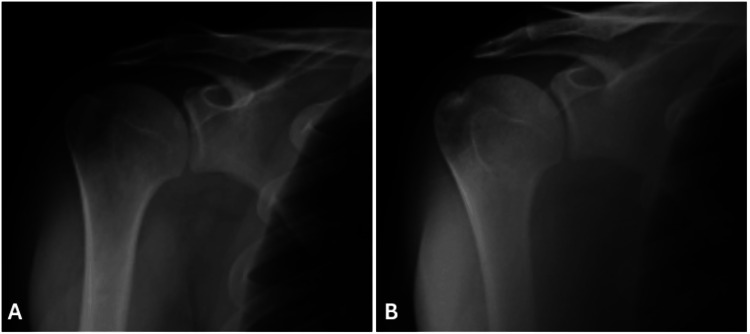
The true anteroposterior radiographs taken immediately post-trauma (A) and at the three-month follow-up (B) demonstrate a normal subacromial space (measured at approximately 10mm).

Under diagnostic ultrasound guidance, 5cc of prilocaine was injected into the subacromial bursa. One hour after the injection, the patient's forward and lateral elevation improved from 150 degrees to 180 degrees, and the pain subsided. The diagnosis was confirmed as subacromial impingement due to subacromial bursitis. The subacromial impingement was not attributed to bony structures, as the direct radiographs demonstrated a normal subacromial space (Figure 4). 

The patient was advised to restrict overhead activities and was prescribed diclofenac sodium 75 mg slow-release tablets to be taken orally twice a day, as well as nimesulide and capsaicin gel for topical application three times a day after ice application. Physical rehabilitation focusing on strengthening the rotator cuff muscles was continued. At the six-month follow-up, the patient had mild pain but a range of motion of 180 degrees. At the one-year follow-up, the patient had a full range of motion without pain.

## Discussion

Approximately 20% of all proximal humerus fractures involve GT fractures, which can often be successfully treated without surgery [8,9]. Catalan has indicated that isolated GT fractures may occur due to rotator cuff avulsion or anterior shoulder dislocation [10]. In our patient, both avulsion and anterior shoulder dislocation mechanisms were involved as the patient was left hanging in the air. The GT is the anatomical footprint region for the rotator cuff. Generally, in proximal humerus fractures, a displacement of more than 10 mm or an angulation of more than 45 degrees requires surgical intervention. However, for GT fractures, a displacement of 5 mm is considered the threshold for surgical treatment [7,9]. Recently, some authors have suggested that even a 3 mm displacement in GT fractures may require surgery [9,11]. Conservative treatment outcomes are shown to be worse for GT fractures displaced posterosuperiorly compared to those displaced in other directions [12]. Additionally, in cases with 3-5 mm displacement, superior displacement of the GT can disrupt rotator cuff biomechanics, leading to subacromial impingement [8]. Rouleau et al. have recommended surgery in such cases [8]. Studies have also indicated that even a 2 mm superior displacement can cause subacromial impingement in GT fractures [4,5]. Catalan has noted that the main potential adverse outcome of displaced GT fractures is impingement in the subacromial region or rotator cuff dysfunction due to associated rotator cuff damage [10]. In our case, the patient had both a GT fracture and a shoulder dislocation. Surgery was not considered due to the absence of displacement. However, the development of subacromial impingement despite the lack of displacement or superior migration is noteworthy. Janssen et al. have demonstrated that in cases where displacement is minimal on plain radiographs, a CT scan does not provide additional value, and direct radiography is sufficient [7]. In our case, a CT scan was performed not due to uncertainty about the degree of displacement but to ensure that no glenoid defect was missed, as the patient had both a GT fracture and shoulder dislocation. Hodgson et al. have shown that early mobilization in proximal humerus fractures results in faster and more successful outcomes, reduces pain, and does not cause additional complications [13]. In line with this, pendulum exercises were initiated in our patient after the second week to prevent frozen shoulder complications. Contrary to expectations, the patient’s pain did not gradually decrease. From the sixth week onwards, TENS and physical therapy exercises were added to pendulum exercises for pain management and rehabilitation. TENS and ice application have been recommended in the literature for pain control during the acute injury phase or post-exercise [14]. A similar protocol was followed in our patient, with TENS and ice applications used for pain control. In the third month post-surgery, persistent pain and restriction in forward elevation led to the diagnosis of subacromial bursitis and impingement via diagnostic ultrasound and local anesthetic injection.

Garving et al. have stated that the main goal of treatment for subacromial impingement is pain relief and restoration of joint function [15]. If there is no major structural damage, conservative treatment for 3-6 months is recommended as the first-line treatment. Initially, pain management, followed by passive and active ROM exercises and ultimately strengthening exercises, are suggested. Additionally, restricting the overhead activities of the affected arm is recommended. In our patient, similar interventions led to dramatic improvement in symptoms within three months. A complete recovery without the need for surgery was achieved by the first year. Garving et al. have also recommended corticosteroid injections with local anesthetics [15]. In the literature, corticosteroid injections are indicated as the standard treatment protocol with Level 1 evidence within the first eight weeks for reducing acute pain and improving shoulder ROM [16,17]. In our young patient, to avoid tendon and soft tissue damage and to protect cartilage tissue, a diagnostic local anesthetic was administered into the subacromial bursa instead of corticosteroids. While corticosteroid application could have potentially resulted in faster recovery, we demonstrated that complete recovery could be achieved without it. If the patient’s symptoms had persisted, an intrabursal corticosteroid injection would have been the initial approach.

## Conclusions

This case report demonstrates that isolated greater tuberosity fractures and associated glenohumeral joint dislocations can be successfully managed with conservative treatment and appropriate rehabilitation approaches. The conservative treatment and rehabilitation protocols applied to our patient resulted in significant improvement of symptoms, achieving a full range of motion and a pain-free state after one year. By the third month, MRI findings had shifted focus away from labral pathologies to subacromial bursitis. During the treatment process, early diagnosis of post-traumatic subacromial impingement syndrome and the use of appropriate rehabilitation methods positively influenced the patient's recovery. Had the patient's pain and movement restrictions persisted, a steroid injection targeting subacromial bursitis and the resultant subacromial impingement syndrome might have been recommended. However, due to the patient's youth, previous swimming experience, and sufficient rotator cuff muscle strength, the potential complications of the steroid injection led to its postponement. Instead, overhead activity restriction, NSAIDs (nonsteroidal anti-inflammatory drugs), and appropriate rehabilitation eliminated the need for a steroid injection.
